# The visible face of intention: why kinematics matters

**DOI:** 10.3389/fpsyg.2014.00815

**Published:** 2014-07-24

**Authors:** Caterina Ansuini, Andrea Cavallo, Cesare Bertone, Cristina Becchio

**Affiliations:** ^1^Department of Robotics, Brain and Cognitive Sciences, Italian Institute of TechnologyGenova, Italy; ^2^Department of Psychology, Centre for Cognitive Science, University of TorinoTorino, Italy

**Keywords:** kinematics, reach-to-grasp, intention, action observation, social interaction

## Abstract

A key component of social understanding is the ability to read intentions from movements. But how do we discern intentions in others’ actions? What kind of intention information is actually available in the features of others’ movements? Based on the assumption that intentions are hidden away in the other person’s mind, standard theories of social cognition have mainly focused on the contribution of higher level processes. Here, we delineate an alternative approach to the problem of intention-from-movement understanding. We argue that intentions become “visible” in the surface flow of agents’ motions. Consequently, the ability to understand others’ intentions cannot be divorced from the capability to detect essential kinematics. This hypothesis has far reaching implications for how we know other minds and predict others’ behavior.

Room H3 in King’s College, Cambridge, was crowded that night. It was 25 October 1946, and Karl Popper and Ludwig Wittgenstein were battling over the very trajectory of their discipline, when Wittgenstein picked up a fire-poker. Did Wittgenstein brandish the poker to threaten Popper, or did he merely pick it up absent-mindedly to give emphasis to his own remarks? (Edmonds and Eidinow, 2001).

When we observe others acting, what matters are their goals and intentions. In the above “poker incident,” what matters – especially from Popper’s point of view – is Wittgenstein’s intention in picking up the poker. But how do we discern intentions in others’ actions? What kind of information about intentions is actually available in the features of others’ movements? (Baldwin and Baird, 2001).

The ability to interpret and predict the behavior of other people hinges crucially on judgments about the intentionality of their actions – whether they act purposefully (with intent) or not – as well as on judgments about the specific intentions guiding their actions. Until recently, however, direct investigation of these skills has been surprisingly rare. One obstacle to such investigation has been the framing of the problem as a problem of access to mental states which are hidden away in the other person’s mind and therefore inaccessible to perception. As Gallagher (2008) puts it, the supposition has been precisely that intentions are “not things that can be seen.”

Recent findings challenge this view by positing that intentions are specified at a tangible and quantifiable level in the movement kinematics (Becchio et al., 2010). “How” an action is performed is not solely determined by biomechanical constraints, but it depends on the agent’s intention, i.e., “why” the action is performed. This raises the intriguing possibility that intentions – regarded as *covert* mental state dispositions by standard theories of social understanding – may become “visible” in a person’s *overt* motor behavior (Runeson and Frykholm, 1983).

In this *Perspective* article, we discuss this hypothesis in light of recent kinematics and psychophysical evidence. An apt characterization of the ability to understand others’ intentions, we argue, may not abstract from a systematic assessment of how intentions translate into movements. In line with this, the first section shows how kinematics techniques can be applied to investigate the influence of intention on grasping movements. Intention is here defined at the level of “why” an actor is performing a specific action with an object, i.e., the distal goal of the action (Grafton and de C Hamilton, 2007). Following the demonstration that intention influences action kinematics, the second section reviews evidence that observers are capable to pick-up intention information from movement patterns. The third and final sections discuss the implications of these findings for future research on action understanding.

## WHAT DOES KINEMATICS TELL US ABOUT INTENTIONS IN ACTION EXECUTION?

**FIGURE 1:**
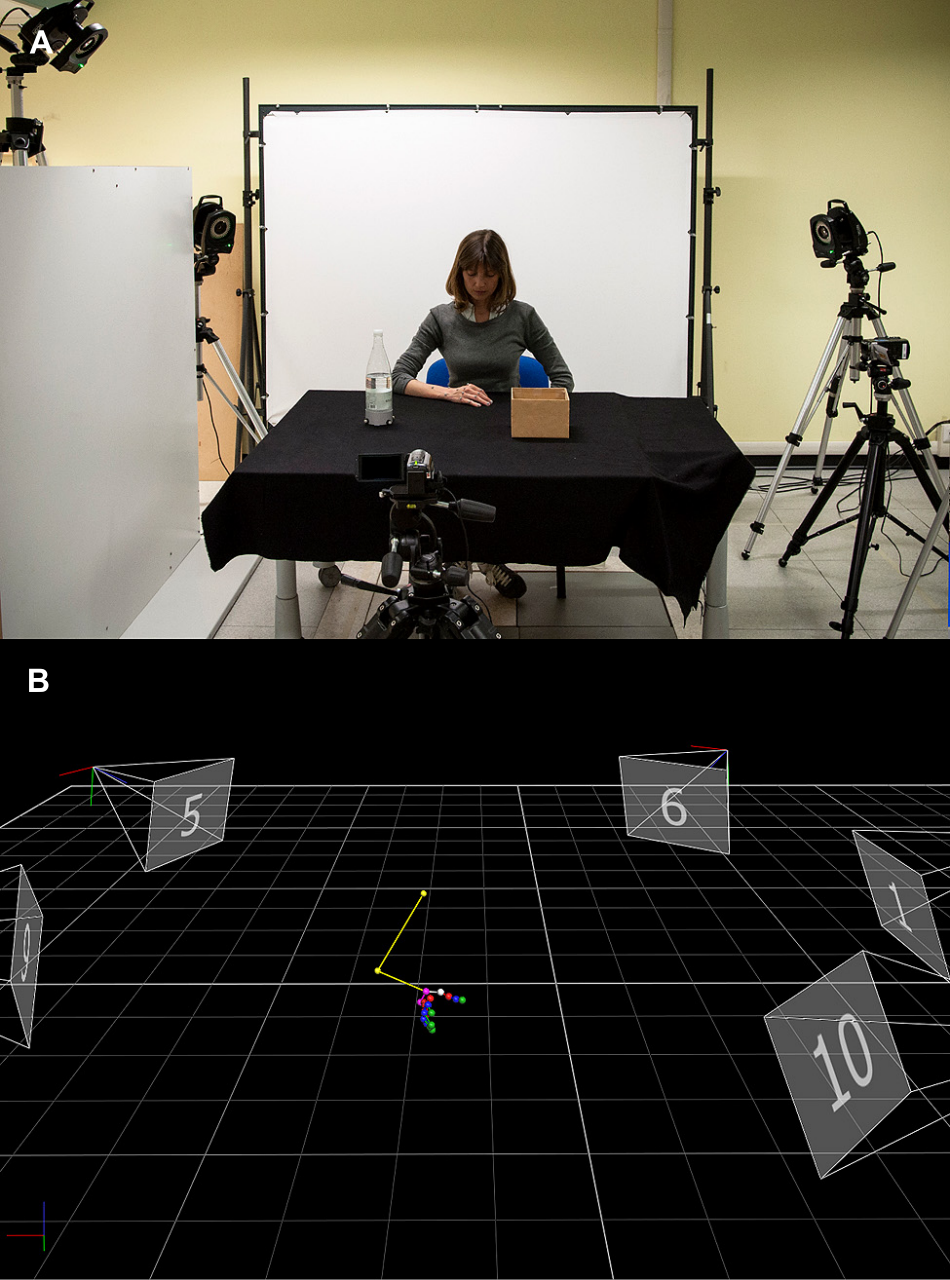
**Techniques used to quantify the influence of intention on movement kinematics. (A)** Example of experimental set-up employed in action execution studies. The participant sits at a table with his hand resting in a starting position, which is kept constant across participants. The task is to reach and grasp the object (i.e., a bottle) either to lift it or to place it inside a box. An optoelectronic system (Vicon Motion Systems Ltd., UK) equipped with nine infra-red cameras is used to quantify reach-to-grasp movements. This system relies on passive markers (retro-reflective material on a plastic sphere) placed on points of interest over participant’s hand. An infra-red light is transmitted toward the work space area and the rays are reflected back off the markers to a series of “cameras” that record their positions. These positions are then referred to a coordinate system, the origin of which is either in 2-D or 3-D coordinates, i.e., two or three mutually orthogonally axes, each passing through the origin. **(B)** A computer-generated stick figure representing the position of the markers placed over arm and hand joints during a reach-to-grasp movement toward the bottle. After collecting raw data, it is possible to identify and track the marker’s trajectories almost in real time by means of tracking procedures.

Research on hand kinematics has proven insightful in revealing how specific kinematic landmarks modulate with respect to object properties, including object size, shape, texture, fragility, and weight. As recently reviewed, all these factors influence the kinematics of grasping (Castiello, 2005). The way an object is grasped, however, does not only depend exclusively on the properties of the object, but it is also influenced by the agent’s intention. This was first demonstrated by Marteniuk et al. (1987) by asking participants to grasp a disk and either fit it carefully or throw it. The *deceleration time* was longer for fitting than for throwing (see **Table 1**). Since this seminal work, a plethora of studies have investigated how intentions influence the execution of reach-to-grasp movements (e.g., Ansuini et al., 2006, 2008; Armbrüster and Spijkers, 2006). The logic of these studies has been to “manipulate” the intention while keeping the object to be grasped (i.e., goal) as well as the situational requirements (i.e., context) constant (see **Figure 1**). If within the same context, the same object is handled differently depending on the agent’s intention, this would indicate that the intention influences the grasping kinematics.

**Table 1:** A brief overview of the main kinematic variables traditionally used to describe reach-to-grasp movements.

	Kinematics variables	Frequently used definition	Units
Proximal component	Wrist velocity	The module of rate of change of marker displacement with respect to time	mm/s
	Reach onset	Time at which the wrist velocity crosses a threshold (e.g., 5 mm/s) and remains above it for a given period (e.g., longer than 500 ms)	ms
	Reach offset	Time at which the wrist velocity crosses a threshold (e.g., 5 mm/s) and remains below it for a given period (e.g., longer than 500 ms)	ms
	Movement duration	Time interval between reach onset and offset	ms
	Time to peak velocity	The moment in time in which the wrist velocity reaches its maximum during movement	ms
	Wrist acceleration	The module of rate of change of velocity with respect to time	mm/s^2^
	Deceleration peak	The moment in time in which wrist acceleration reaches the minimum; it occurs between time to peak velocity and reach offset	ms
	Acceleration peak	The moment in time in which wrist acceleration reaches the maximum; it occurs between reach onset and time to peak velocity	ms
Distal component	Grip aperture	The Euclidean distance between the marker placed on thumb tip and that placed on the tip of the index finger	mm
	Time to max grip aperture	The moment in time when the maximum distance between the thumb and the index finger was reached during movement	ms
	Grip aperture velocity	The rate of change of the grip aperture with respect to time	mm/s

*The proximal component refers to the “reaching” and is described by variables obtained from the radial aspect of the wrist. The distal component refers to the “grasping” and is described by variables obtained from thumb and index fingers. With three or more markers (a configuration classically used for reach-to-grasp movements), the distances and angles at joints can be measured as well as the accelerations and velocities of hand and limb segments. Please note that to compare movements with different absolute durations, time variables can be normalized with respect to the movement duration (e.g., % of normalized movement duration).*

This hypothesis has been tested in two-digit grasp studies as well as in multi-digit grasp studies that investigated how the whole hand is shaped during the unfolding of the reach-to-grasp movement. Ansuini et al. (2008), for example, asked participants to reach toward and grasp a bottle to accomplish one of four possible actions: pouring, displacing, throwing, or passing. Analysis of digit kinematics revealed that when the bottle was grasped with the intent to pour, both the middle and the ring fingers were more extended than in all the other considered intentions. Similarly, choice of hand placement on the object has been shown to adapt to the upcoming intention. For example, participants place their thumb and index finger in a higher position when they grasp a bottle with the intention to pour than when they grasp it with the intention to lift (Crajé et al., 2011).

Further studies have extended these effects to the domain of social intention. For instance, it has been shown that participants’ *maximal finger aperture* is smaller and *grip aperture velocity* increases when an object is reached and grasped with the intent to move it compared to when it is grasped with the intent to pass it to another person (Becchio et al., 2008a; see also Sartori et al., 2009; Quesque et al., 2013). At a higher level of abstraction, Becchio et al. (2008b; see also Georgiou et al., 2007) showed that the kinematics of grasping movements differed depending on whether the object was grasped with the intent to cooperate with a partner, compete against an opponent, or perform an individual movement at slow or fast speed. Despite similar task requirements, *movement duration* was shorter and *wrist velocity* was higher for “competitive” than for “individual fast” movements. Conversely, *movement duration* was longer and *wrist velocity* was lower for “cooperative” than for “individual slow” movements.

## WHAT DOES KINEMATICS TELL US ABOUT INTENTIONS IN ACTION OBSERVATION?

The above findings suggest that intentions influence action planning so that, although the to-be-grasped object is the same, different kinematic features are selected depending on the overarching intention. That intention information is available in the kinematic pattern of human action, however, is not to say that it can be perceptually appreciated. Are observers sensitive to differences in movement kinematics*?* Can they use them to discriminate between movements performed with different intentions?

One approach for probing the contribution of visual kinematics is progressive temporal occlusion, where multiple occlusion points are used so as to provide selective vision to different time periods or events within an observed action sequence (Farrow et al., 2005). This paradigm has been used with a number of different sports to demonstrate superior attunement to advance kinematic information by experts over non-experts (e.g., Abernethy and Zawi, 2007; Abernethy et al., 2008). For example, it has been shown that in racquet sports such as badminton to predict the depth of an opponent’s stroke, expert players use advance pre-impact kinematic information to which less skilled players are not attuned (Abernethy and Zawi, 2007).

Adapting the same logic to intention anticipation, Sartori et al. (2011) tested whether observers use pre-contact kinematic information to anticipate the intention in grasping an object. To this end, they first analyzed the kinematics of reach-to-grasp movements performed with different intents: cooperate, compete against an opponent, or perform an individual action at slow or fast speed. Next, they selected videos representative of each type of intention and prepared experimental video-clips. Each clip started before reach onset and ended at the time the fingers contacted the object so that neither the second part of the movement, nor the interacting partner, when present, were visible. Participants watched these videos and judged the intention in a yes/no detection task. The results revealed that observers were able to judge the agent’s intent by simply observing the initial reach-to-grasp phase of the action (Sartori et al., 2011; but see also Naish et al., 2013).

But what specific cues did participants use to make their anticipation judgments? To examine the spatial location of anticipatory information, in a second psychophysical study, Sartori et al. (2011) combined temporal and spatial occlusion procedures to mask visibility to selected spatial areas of the agent’s movement. Masking the visibility of the upper part of the agent’s body (i.e., from shoulders to head) caused no significant decrements in prediction accuracy, suggesting that observers were able to pickup useful information from the arm kinematics (Sartori et al., 2011).

The spatial occlusion method helps to determine how much information is lost when a specific spatial region of the display is masked. However, because other areas of the display can potentially provide compensatory or alternative information, it does not indicate how much information is carried in isolation by specific kinematic features (Abernethy et al., 2008). To obtain an analytic determination of the key kinematic features that provide useful advance information about the agent’s intention, in a subsequent study Manera et al. (2011) rendered reach-to-grasp movements as point-light displays. Though the displays were reduced to only three disconnected points of light corresponding to the position of the markers on the wrist, the index finger, and the thumb of the agent’s hand, participants were nonetheless able to discriminate between social and individual intentions from the unfolding movement kinematics.

## UNDERSTANDING OTHERS’ INTENTIONS: IMPLICATIONS AND FUTURE DIRECTIONS

Considered together, the studies reviewed above indicate that observers are capable of picking up and using kinematic information to make judgments not only about movement patterns but also about intentions. In this section, we consider some of the theoretical and the methodological issues raised by these findings and speculate on the ways in which they may be addressed by future research.

### HOW DOES KINEMATICS COMBINE WITH OTHER SOURCES OF INFORMATION?

How does movement kinematics combine with other sources of information in revealing others’ intentions? There are situations in which the intention of an observed actor can be unambiguously estimated from one source of information, e.g., the type of grasp, the presence of a target object. Most often, however, combining different sources of information may lead to more accurate predictions. This is indeed what Stapel et al. (2012) demonstrated by asking participants to anticipate how an observed action would unfold. Participants observed an actor walking. After a few steps, they had to indicate how the action would continue, i.e., whether the actor would take another step walking or start crawling. A first experiment showed that observers were more accurate when they could base their predictions on the combination of movement kinematics, situational constraints (e.g., the presence of a table), and target object position (a ball). In a second experiment, the target object was artificially moved to another location so that movement kinematics was incongruent with the target object position. Results revealed that, in this ambiguous situation, participants relied on movement kinematics rather than on object location in making their predictions. This suggests that in the presence of conflicting information from different sources, movement kinematics may be prioritized to disambiguate the agent’s intention. A challenge for future research will be to understand the temporal course of information integration from different sources. A recent transcranial magnetic stimulation (TMS) study by Cavallo et al. (2013) demonstrated that, at movement onset, motor-evoked potential responses reflected the most probable motor program estimated from the situational context (e.g., whole hand grasp). During movement observation, however, the initial motor program was substituted by a new plan matching the specific features of the observed movement (e.g., precision grip). Thus, an intriguing possibility is that the contribution of movement kinematics is related to the specific stage of the observed action processing: before the to-be-observed action starts, observers rely on contextual factors to predict the course of the action; as the movement unfolds, however, action prediction might prioritize kinematic information. If confirmed, this would have implications for the interpretation of the so-called chain model of action organization (Bonini et al., 2013): modulation of mirror neuron discharge by end-goal might reflect not only (and not so much) the presence of contextual cues allowing the monkey to predict the experimenter’s intention (Fogassi et al., 2005), but also sensitivity to intention-related differences in the movement kinematics.

### “SECOND-PERSON” vs. “THIRD-PERSON” INTENTION UNDERSTANDING

The studies reviewed above used spatial and temporal occlusion procedures to quantify pick-up of advance information. The advantage of using psychophysical methods is the high degree of control and statistical power they ensure. However, it is not clear how far this type of paradigm accounts for real-time interactions in which two or more individuals are set in a common social context. Social cognition has been proposed to be substantially different when we actively interact with others (“second-person” social cognition) rather than merely observe them (“third-person” social cognition; Schilbach et al., 2013). For third-person social cognition, observing body movement is merely a way of gathering data about the other person. For second-person social cognition, the knowledge of the other resides – at least in part – in the interaction dynamics “between” the agents (De Jaegher et al., 2010); it is thus plausible that interaction dynamics affect pick-up and use of advance kinematic information.

An initial investigation on this topic was made by Streuber et al. (2011) by adapting the spatial occlusion procedure to a social interaction task. Participants played a table tennis game in a dark room with only the table, the net, and the ball visible. The game could be played in a cooperative fashion, i.e., to play the ball back and forth as often as possible, or in a competitive fashion, i.e., to win the trial. The visibility of the players’ racquets and the body movements was manipulated with the following logic. If a specific source of information is important for playing table tennis, then rendering this source of information visible should positively affect the players’ performance. Results revealed that when the game was played cooperatively, seeing the other player’s racket had the largest effects on performance. In contrast, when the game was played competitively, seeing the other player’s body resulted in the largest increase in performance. This suggests that online cooperative and competitive dynamics selectively modulates the use of visual information about others’ actions. A question to be addressed by future research is whether a similar modulation is observed in oﬄine tasks, in which participants are required to merely observe cooperative and competitive actions. More generally, it would be interesting to directly compare second-person and third-person social understanding with respect to the pick-up and the use of advance information: is attunement to kinematic features modulated by self-involvement? Do second-person and third-person intention understanding rely on the same kinematic characteristics?

### WHAT IS THE NATURE OF THE MECHANISMS WHICH ALLOW US TO READ INTENTIONS IN OTHERS’ ACTIONS?

Ever since their discovery, mirror neurons have been proposed to underlie our ability to understand actions “transforming visual information into knowledge” about others’ goals and intentions (Gallese and Goldman, 1998). But how exactly is this transformation achieved?

Rizzolatti and Craighero (2004) suggested a rather simple mechanism: “Each time an individual sees an action done by another individual, neurons that represent that action are activated in the observer’s premotor cortex.” This motor representation of the observed action “corresponds to that which is spontaneously generated during active action and whose outcome is known to the acting individual.” In this way, mirror neurons would transform visual information into knowledge about another person’s intention.

This model has been criticized on the assumption that “the same visual kinematics can be caused by different goals and intentions” (Kilner et al., 2007). Simulating the observed kinematics – it has been claimed – might allow an observer to represent what the agent is doing. However, given the non-specificity of the observed kinematics, it will not allow them to represent the agent’s intention (Jacob and Jeannerod, 2005).

The findings reviewed above provide strong evidence to the contrary. First, in contrast to the “non-specificity assumption,” they demonstrate that intention information is specified in the visual kinematics. Second, they indicate that observers are sensitive to this information and can use it to discriminate between different intentions. Evidence that the mirror system supports this ability comes from recent fMRI studies (Vingerhoets et al., 2010; Becchio et al., 2012). For example, Becchio et al. (2012) report that mirror areas are sensitive to kinematic cues to social intention. Participants observed isolated reach-to-grasp movements performed with the intent to cooperate, compete, or perform an individual movement, followed by a static test picture. They were required to judge whether the test picture depicted a continuation of the observed movement or not. Despite the lack of contextual information, observing grasping movements performed with a social intent relative to grasping movements performed with an individual intent activated mirror areas, including the inferior frontal gyrus and the inferior parietal lobule. Interestingly, comparison of social vs. individual movements also revealed differential activations at the temporo-parietal junction and within the dorsal medial prefrontal cortex, two regions traditionally associated with explicitly thinking about the state of minds of other individuals (i.e., “mentalizing”). These findings shed some light on the neural mechanisms underlying intention-from-movement understanding. They leave, however, a number of crucial issues unanswered.

A first issue pertains to how observed actions are mapped onto one’s own motor system. The mirror system is generally assumed to associate observed actions with “corresponding” motor programs of the observer. What though is exactly meant by “corresponding?” When we observe other individuals act, the very fact that our body differs from theirs’ introduces a disparity between the observed and the executed kinematics (for data on this issue see for instance Gazzola et al., 2007). It is thus difficult to envision how, at a computational level, the executed kinematics might be “coupled” with the observed kinematics (but see Press et al., 2011).

A second question concerns the exact contribution provided by the mirror and the mentalizing system (Van Overwalle and Baetens, 2009). While some theorists have argued that these two systems are mutually independent (e.g., Jacob and Jeannerod, 2005; Saxe, 2005), a substantial number of authors support the notion that the mirror system might inform the mentalizing system (e.g., Keysers and Gazzola, 2007; Uddin et al., 2007). According to this view, people would use their own motor system to encode the intentionality of an action based on its visual properties and form a pre-reflective representation of the other person’sintention. This representation would then serve as inputs to attributional processing within the mentalizing system (Keysers and Gazzola, 2007; see also Spunt and Lieberman, 2012). In line with this, de Lange et al. (2008) report that mirror areas, including the inferior frontal gyrus, process the intentionality of an observed action on the basis of the visual properties of the action, irrespective of whether the subject paid attention to the intention or not. In contrast, brain areas that are part of the mentalizing network become active when subjects reflect about the intentionality of an observed action, but are largely insensitive to the visual properties of the observed action. Alternatively, mirror neurons might discharge during action observation not because they are driven by the visual input but because they are part of a generative model that is predicting the sensory input (Kilner, 2011). Within this framework, the generative model starts with a prior prediction of the intention of the observed action. This prediction would be estimated in areas outside the mirror system (including mentalizing areas) and then conveyed to mirror areas, influencing the selection of a specific action intention. Techniques for characterizing effective connectivity between brain areas can provide answers in this debate because they can demonstrate the influence one system exerts over the other.

## CONCLUSION

The view that “motor” is separated from “mental” has long been dismissed, yet traces of it remain in the way the problem of intention understanding is currently addressed. Based on the assumption that intentions are hidden away and therefore not accessible to perception, standard theories of social cognition have mainly focused on the contribution of higher level, inferential processes to intention understanding. We argue that reframing the relationship between intention and movement provides radically new insights into the psychology and neurobiology of how we know other minds and predict others’ behavior.

Did Wittgenstein pickup the poker to threaten Popper or to give emphasis to his thoughts? As Popper’s account of the episode proves, the way in which Wittgenstein brandished the poker clearly betrayed his intention.

## Conflict of Interest Statement

The authors declare that the research was conducted in the absence of any commercial or financial relationships that could be construed as a potential conflict of interest.
